# Association between serum levels of GDF-15, suPAR, PIVKA-II, sdLDL and clinical outcomes in hospitalized COVID-19 patients

**DOI:** 10.1007/s11739-024-03630-7

**Published:** 2024-05-03

**Authors:** Alessio Molfino, Emanuela Anastasi, Eleonora Assanto, Ludovica Toccini, Giovanni Imbimbo, Antonietta Gigante, Valentina Viggiani, Antonella Farina, Orietta Picconi, Antonio Angeloni, Maurizio Muscaritoli

**Affiliations:** 1 Department of Translational and Precision Medicine, Sapienza University of Rome, Rome, Italy; 2 Department of Experimental Medicine, Sapienza University of Rome, Rome, Italy; 3 National HIV/AIDS Center, Istituto Superiore Di Sanità, Rome, Italy; 4 Department of Molecular Medicine, Sapienza University of Rome, Rome, Italy

**Keywords:** COVID-19, Inflammation, Internal medicine, Prognosis, Biomarkers, GDF-15, suPAR, PIVKA-II, sdLDL

## Abstract

To quantify the circulating levels of novel serum biomarkers including GDF-15, PIVKA-II, sdLDL, suPAR, and of CRP in hospitalized COVID-19 patients compared with healthy subjects, and to evaluate their association(s) with outcomes in COVID-19. We considered patients with confirmed COVID-19, hospitalized in an Internal Medicine ward. The clinical characteristics were collected, including the number and type of comorbidities. Serum levels of GDF-15, PIVKA-II, suPAR, sdLDL, as well as CRP were measured. As outcomes, we considered Intensive Care Unit (ICU) transfer or death, as well as the length of stay (days) and in-hospital complications. Data were statistically analyzed, as appropriate, and a *p* value < 0.05 was considered significant. Ninety-three patients and 20 healthy controls were enrolled. COVID-19 patients vs. controls showed higher median levels of GDF-15 (*p* < 0.0001), PIVKA-II (*p* < 0.0001) and sdLDL (*p* = 0.0002), whereas no difference was observed for suPAR. In COVID-19 patients, the most frequent comorbidities were arterial hypertension (62.4%) and cardiovascular disease (30.1%). GDF-15 levels positively correlated with age (*r* = 0.433, *p* < 0.0001), and this correlation was confirmed for suPAR (*r* = 0.308, *p* = 0.003) and CRP (Rho = 0.40 *p* < 0.0001), but not for PIVKA-II and sdLDL. Higher GDF-15 levels were associated with a higher number of comorbidities (*p* = 0.021). The median length of stay was 22 (15; 30) days. During hospitalization, 15 patients (16%) were ICU transferred, and 6 (6.45%) died. GDF-15 serum levels correlated with the length of stay (rho = 0.27 *p* = 0.010), and were associated with ICU transfer or death (*p* = 0.003), as well as PIVKA-II (*p* = 0.038) and CRP (*p* < 0.001). Moreover, higher GDF-15 and PIVKA-II serum levels were associated with infectious complications (*p* = 0.008 and *p* = 0.017, respectively). In this cohort of hospitalized COVID-19 patients, novel inflammatory biomarkers, including GDF-15, suPAR and PIVKA II were associated with some patient’s clinical characteristics, complications, and poor outcomes.

## Introduction

COVID-19 has been a global emergency and still represents a health-care problem worldwide since the last 4 years [1]. Because of the extremely relevant clinical and social impact of COVID-19, several studies have been conducted on its clinical manifestations, diagnostic and predictive variables, as well as novel therapies and their impact on outcomes [2, 3]. One of the main clinical features characterizing COVID-19, especially during the acute phases, is represented by enhanced inflammatory status [4].

In particular, during the first waves of the pandemic, especially before vaccination campaigns, when the grade of inflammation was extremely high, patients have been more frequently hospitalized in intensive care settings [4, 5]. Inflammatory status has been linked to increased oxygen need [6], as well as with the use of non-invasive ventilation, infections [7] and cardiovascular complications [2]. Often the multi-organ involvement had a rapid course and was unexpected. In this light, data indicated that the assessment of inflammatory biomarkers at hospital admission could early identify patients with higher risk to develop a severe form of COVID-19 [8]. Moreover, COVID-19 may differ from other infectious diseases in terms of modulation of inflammatory biomarkers, especially in comparison with bacterial sepsis. However, how bacterial sepsis parallels and/or differs from COVID-19 in terms of biomarkers is still unclear [9].

In particular, high C-reactive protein (CRP) levels have been extensively described in COVID-19, being associated with worse prognosis [10], although it remains a marker with low specificity.

Recent data showed that growth differentiation factor-15 (GDF-15), a cytokine of transforming growth factor (TGF)-β superfamily, was involved in acute inflammatory conditions [11]. In COVID-19 setting, higher GDF-15 plasma levels have been associated with lower SpO2/FiO2 ratio, and therefore with pulmonary dysfunction [12].

Moreover, suPAR, the soluble form of urokinase-type plasminogen activator receptor (uPAR), a glycosyl-phosphatidylinositol (GPI)-linked membrane protein, was associated with inflammation and immune activation [13]. In patients with COVID-19, suPAR levels were associated with the risk of venous thromboembolism [14].

The state of hypercoagulability has been also related with the levels of prothrombin induced by vitamin K deficiency or antagonist-II (PIVKA-II), which is a marker of vitamin K status [15]. Importantly, vitamin K was also described to be involved in anti-oxidative pathways, as well as immune and inflammatory modulation [16].

In addition, inflammation linked to cardiovascular morbidity has been extensively described in COVID-19 patients, who showed increased small dense Low-Density Lipoprotein (sdLDL) levels when developing ischemic stroke and/or cardiovascular complications [17, 18].

However, data on the involvement of these biomarkers in the early phase (at admission) of COVID-19 in non-ICU setting are limited or unknown, especially they were not tested all together within the same cohort of patients.

In the present study, we aimed to quantify the circulating levels of novel serum biomarkers including GDF-15, PIVKA-II, sdLDL, suPAR, and of CRP in hospitalized COVID-19 patients compared with healthy subjects and to evaluate their association(s) with outcomes in COVID-19.

We then analysed the association(s) between the levels of these biomarkers with patients’ clinical characteristics.

## Material and methods

### Participant’s selection and clinical variables

We included in this single-center study, adults (≥18 years) patients with confirmed COVID-19 disease hospitalized in non-ICU setting, who did not require mechanical ventilation, consecutively admitted from December 2020 to May 2021 to the Internal Medicine ward for COVID-19 patients, Azienda Policlinico Umberto I Hospital, Sapienza University of Rome, Italy.

Inclusion criteria were the age ≥ 18 years and the ability to sign informed consent.

The diagnosis was performed by nasopharyngeal or oropharyngeal swab (positive real-time reverse-transcriptase polymerase chain reaction—RT-PCR). The Ethics Committee of Azienda Policlinico Umberto I, Sapienza University of Rome approved the study (protocol n.109/2020). The patients signed the informed consent. All COVID-19 cases were recorded, and the data forwarded to the National Health Ministry, as required by Italian regulations.

Standard laboratory techniques were used for testing haemoglobin, white blood cells, serum albumin, C-reactive protein (CRP; normal values: 0–5000 mcg/l), D-dimer, ferritin and creatinine. We collected data on demographic variables (age and sex) and medical history, including comorbidities and medications. All the patients received the standardized therapy available at that time for COVID-19 [19].

In all the participants, we collected blood samples and stored serum of each patient at −80 °C for biomarkers assessment.

### Serum biomarkers in COVID-19 patients and in controls

The serum samples were separated and frozen from whole blood within two hours post-draw.

All the samples were measured in duplicate, and the mean value of at least two measurements was used for the analyses in COVID-19 patients, as well as among healthy subjects, represented by blood donors, at our study site, not reporting comorbidities, serving as controls.

### GDF-15

Serum GDF-15 levels were detected by enzyme-linked immunosorbent assay (ELISA) (Quantikinine Quickit Elisa, R&D Systems- Minneapolis, USA). The intra and inter-assay CV were respectively <3.0% and <10%.

### PIVKA-II

PIVKA-II serum values were measured on a Lumipulse G1200 (Fujirebio-Europe, Gent, Belgium), using the LUMIPULSE G PIVKA-II kit (Fujirebio, Tokyo, Japan) a full automated instrument based on elettrochemiluminescence (CLEIA) technology. The intra and inter-assay CV were respectively <2.4% and <10%.

### sdLDL

Serum sdLDL levels were detected by a Sandwich-ELISA kit (Novus 12 Cambridge Science Park, Milton Road, Cambridge, CB4 0FQ, UK. The intra and inter-assay CV were respectively <2.8% and <10%.

### suPAR

Serum levels of suPAR were measured with the human suPARnostic ELISA kit (Biorbyt Ltd. 7 Signet Court, Swann’s Road, Cambridge, CB5 8LA, United Kingdom). The intra and inter-assay CV were respectively <2.7% and <10%.

### Outcomes

We registered the following in-hospital complications: (i) number and type of infections (e.g., urinary tract, pulmonary superinfections, sepsis, septic shock); (ii) cardiovascular events (i.e., thromboembolism, and acute ischemic events, arrhythmias).

The other outcomes evaluated were ICU transfer for worsening of the clinical conditions requiring non-invasive ventilation (NIV) or intubation, length of stay (days), and number of deaths during hospitalization.

### Statistical analyses

We described the participant’s characteristics using mean ± standard deviation and median (25th and 75th percentile) for continuous normally and non-normally distributed variables, respectively. Normal distribution was tested using the Shapiro Wilk test. Categoric variables were shown as number (%). We used the *t*-test or Mann–Whitney, according to normal or non-normal distribution, to evaluate differences between groups and the biomarkers were logarithm transformed (natural logarithm, ln) to reduce skewness when assessing their association with outcomes. The correlations between variables were verified by Pearson’s test or Spearman’s test, as appropriate. Association between categorical variables were assessed by Chi-square test.

Receiver Operating Characteristics (ROC) curves of the different serum biomarkers were performed to evaluate their association with poor outcome (ICU transferred or death) and infectious complications.

Considering the absence of data on these novel biomarkers in COVID-19 patients compared to controls, we performed a post-hoc power analysis that resulted greater than 90% given the number of participants in the two groups (93 COVID-19 patients and 20 controls), their serum biomarkers levels, and an alpha = 0.05 by using the GPower 3.1 software. A *p*-value < 0.05 was considered statistically significant. SPSS version 26 and STATA 8.2™ were implemented to perform statistical analyses.

## Results

### Participant’s characteristics

We enrolled a total of 93 COVID-19 patients (51 women) with a mean age of 67.6 ± 16.3 years. Body mass index (BMI, kg/m^2^) was within normal range (18.5–24.9) in 49 patients (52.7%), indicating underweight condition (<18.5) in 9 patients (9.7%) and overweight or obesity (≥25) in 35 patients (37.6%) (Table 1). The most frequent comorbidities were arterial hypertension (62.4%), cardiovascular disease (30.1%), including chronic coronary heart disease, chronic heart failure, and atrial fibrillation, diabetes (23.7%), dyslipidaemia (22.6%) (Table 1). Chronic medications included ACE inhibitors, angiotensin II receptor blockers (ARBs), glucose-lowering drugs (i.e., metformin and insulin), statins, and oral anticoagulants. Control group included 9 females and 11 males with a mean age of 61.1 ± 9.5 years. Mean age was not different between patients and controls (*p* = 0.075).

**Table 1:** Patients’ characteristics (*N* = 93)

Parameter	Mean ± SD*
Age, years	67.6 ± 16.3
Females, *n* (%)	51 (54.8%)
BMI classes, *n* (%)	
<18.5 kg/m^2^	9 (9.7%)
18.5–24.9 kg/m^2^	49 (52.7%)
≥25 kg/m^2^	35 (37.6%)
Blood parameters	
Haemoglobin (g/dl)	12.6 ± 2.1
White blood count (mm^3^)	7.2 ± 3.8
Albumin (g/dl)	3.6 ± 0.5
Creatinine (mg/dl)	0.9 (0.74–1.07)*
D-dimer (mcg/l)	858 (573–1652)*
Ferritin (ng/ml)	1374.5 (293.3–1965.8)*
Main comorbidities, *n* (%)	
Arterial hypertension	58 (62.4%)
Cardiovascular disease	28 (30.1%)
Diabetes mellitus	22 (23.7%)
Dyslipidaemia	21 (22.6%)
Outcomes, *n* (%)	
Length of stay (days)	22 (15–30)*
Deaths	6 (6.45%)
ICU transfer	15 (16%)
Infectious complications	37 (40%)
Lung superinfections	21 (22.6%)
Urinary tract infections	13 (14%)
Sepsis	5 (5.4%)
Other infections	6 (6.5%)
Cardiovascular events	10 (11%)

*Median (25th–75th percentile) is indicated for non-normally distributed variables

*BMI* body mass index, *GDF-15* growth differentiation factor-15, *suPAR* soluble form of urokinase-type plasminogen activator receptor, *PIVKA-II* prothrombin induced by vitamin K deficiency or antagonist-II, *sdLDL* small dense low-density lipoprotein, *ICU* Intensive Care Unit

During hospitalization, all patients received the same treatments for COVID-19, based as previously stated on the available guidelines at that time [19], which included intravenous corticosteroids (dexamethasone 6 mg/day), subcutaneous heparin for prophylaxis of thromboembolic events, remdesivir (day 1 loading dose: 200 mg IV, then day 2 and thereafter 100 mg IV qd) and oxygen therapy.

### GDF-15, suPAR, CRP, PIVKA-II and sdLDL in serum of COVID 19 patients and of healthy controls

The serum levels of GDF-15, suPAR, CRP, PIVKA-II and sdLDL in COVID-19 patients and in controls are shown in Table 2. In summary, COVID-19 patients vs. controls showed higher median levels of GDF-15 (pg/ml) (*p* < 0.0001), PIVKA-II (ng/ml) (*p* < 0.0001) and sdLDL (nmol/ml) (*p* = 0.0002), whereas no difference was observed in suPAR (ng/ml) median levels (*p* = 0.153) (Table 2).

**Table 2:** Differences in serum biomarkers between COVID-19 patients (*N* = 93) and healthy controls (*N* = 20)

Biomarker	COVID-19 patients	Controls	*p*-value**
GDF-15 (pg/ml)	760 (510–1115)	364 (193–526)	<0.001
suPAR (ng/ml)	3.9 (1.8–6.7)	2.95 (2.8–3.7)	0.153
PIVKA-II (ng/ml)*	46 (33–86)	26 (23–28)	<0.001
sdLDL (nmol/ml)	4.1 (3.7–4.7)	2.8 (1.5–3.9)	0.002
CRP (mcg/l)	15000 (5400–39200)	1800 (850; 2635)	<0.001

*Data available on 83 COVID-19 patients

**Mann–Whitney test

Data are shown as median values, 25th–75th percentile

The values of these biomarkers were not different between men and women and none of the biomarkers correlated with each other in COVID-19 patients, as well as in controls.

**Fig. 1:**
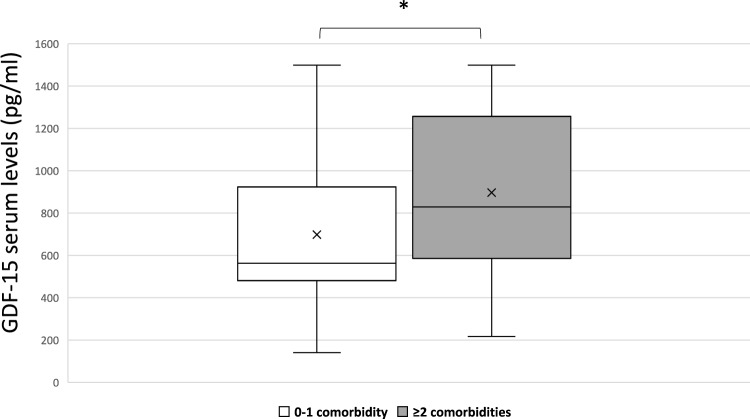
Growth differentiation factor-15 (GDF-15) serum levels in patients with more comorbidities (*n* ≥ 2) vs. those with less comorbidities (*n* ≤ 1) (*p = 0.021). “x” indicates the mean value

**Fig. 2:**
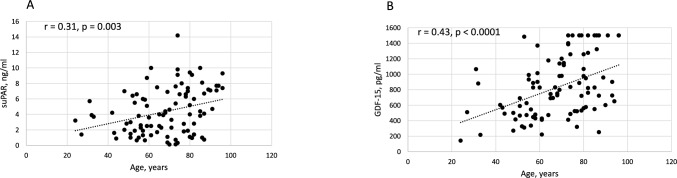
Correlation between soluble urokinase-type plasminogen activator receptor (suPAR) (ng/ml) and age (years) (**A**). Correlation between growth differentiation factor-15 (GDF-15) serum levels (pg/ml) and age (years) (**B**)

In COVID-19 patients, the GDF-15 serum levels resulted higher according to the presence of two or more comorbidities (*p* = 0.021) (Fig. 1). Also, GDF-15 levels positively correlated with age (rho = 0.43, *p* < 0.0001) (Fig. 2A), and the same positive correlation with age was present for serum levels of suPAR (rho = 0.31, *p* = 0.003) (Fig. 2B), as well as for CRP (rho = 0.40, *p* < 0.0001).

### Serum biomarkers and outcomes in COVID-19 patients

#### Length of stay, ICU transfer and mortality

The median length of stay in our cohort was 22 days (15; 30).

Only GDF-15 serum levels significantly correlated with the length of stay (days) (rho = 0.27, *p* = 0.01).

Out of 93 patients, 15 (16%) required ICU transfer for worsening of the respiratory failure, and 6 died (6.45%). Higher GDF-15 levels were associated with a poor outcome (ICU transfer or death) (*p* = 0.003) (Table 3). Also, higher PIVKA-II levels were associated with ICU transfer or death (*p* = 0.038) (Table 3). None of the other biomarkers were associated with poor prognosis.

**Table 3:** Serum levels of GDF-15, PIVKA-II, suPAR, sdLDL and CRP in patients with “positive outcome” and in those with “poor outcome”

Biomarker	Positive outcome^#^ (*N* = 72)	Poor outcome* (*N* = 21)	*p*-value***
GDF-15 (ln, pg/ml)	6.54 (6.17–6.87)	6.88 (6.65–7.31)	*p* = 0.003
PIVKA-II (ln, ng/ml) **	3.83 (3.49–4.25)	4.74 (3.54–5.53)	*p* = 0.038
suPAR (ln, ng/ml)	1.32 (0.52–1.90)	1.55 (0.48–1.98)	*p* = 0.659
sdLDL (ln, nmol/ml)	1.4 (1.28–1.54)	1.4 (1.32–1.58)	*p* = 0.727
CRP (ln, mcg/l)	9.4 ± 1.4	10.7 ± 1	*p* < 0.001

^#^Survived and discharged at home from the Internal Medicine ward

*Intensive Care Unit transferred or death

**Data available on 66 patients of positive outcome group and 17 patients of poor outcome group

***Mann–Whitney test except for T-student test for CRP

Data are shown as median values, 25th–75th percentile except for CRP (mean ± standard deviation)

**Fig. 3:**
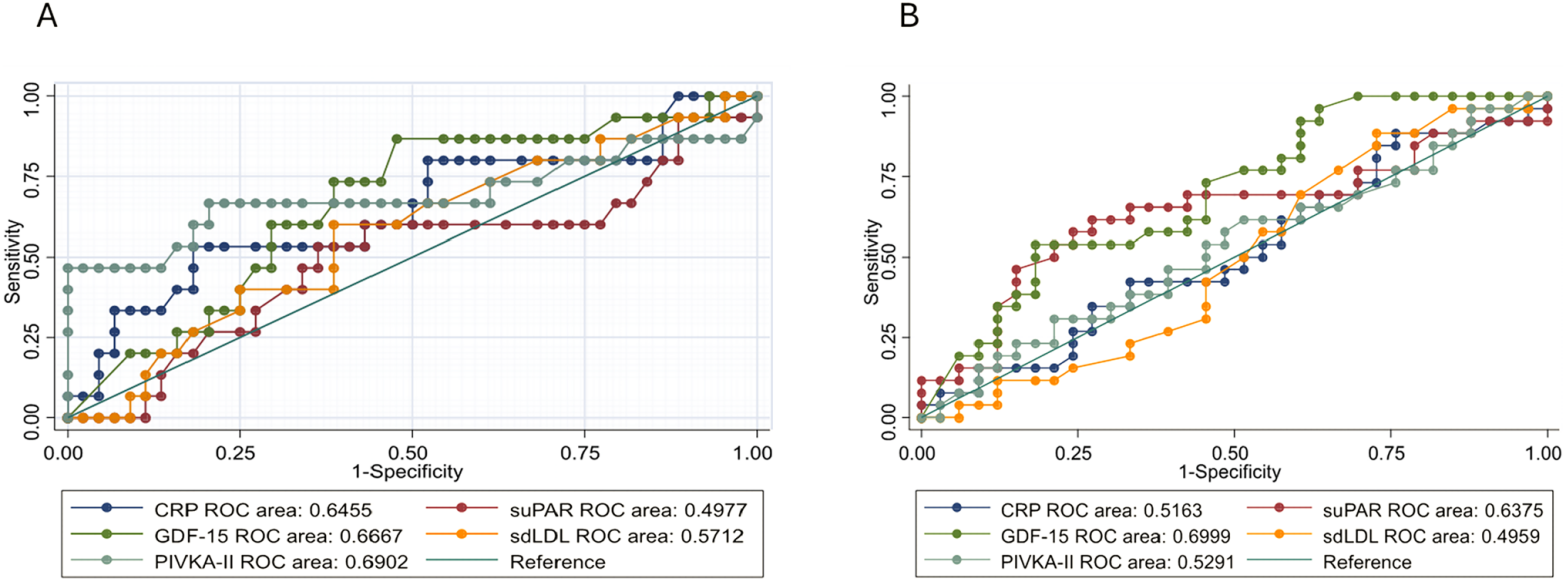
ROC curves of serum levels of GDF-15, PIVKA-II, suPAR, sdLDL and CRP in COVID-19 patients for the prediction of “poor outcome” (Intensive Care Unit transferred or death) (**A**). ROC curves of serum levels of GDF-15, PIVKA-II, suPAR, sdLDL and CRP in COVID-19 patients for the prediction of infectious complications (**B**). *ROC* receiver operating characteristic

In Fig. 3A, we report the area under the curve (AUC) of the biomarkers associated with poor outcomes among COVID-19 patients with abnormal serum CRP values (*n* = 59). Although the AUC among the biomarkers were not statistically different, GDF-15 and PIVKA showed higher AUC.

#### Infectious complications

Out of 93 patients, 37 (40%) were complicated with infections, mainly pulmonary, urinary or systemic (Table 1).

**Fig. 4:**
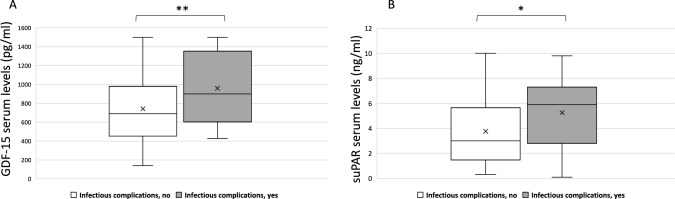
Growth differentiation factor-15 (GDF-15) (**A**) and suPAR serum levels (ng/ml) (**B**) in COVID-19 patients with in-hospital infectious complications vs. those without infectious complications (*p < 0.05; **p < 0.01). “x” indicates the mean value

The mean serum levels of GDF-15 (pg/ml) were significantly higher in patients who developed infectious complications compared with those without infections (959.5 ± 273.1 vs. 742.8 ± 377.5; *p* = 0.008) (Fig. 4A).

Also, serum suPAR (ng/ml) concentrations were higher in those complicated with infections compared with those without (5.3 ± 3.2 vs. 3.8 ± 2.7) (*p* = 0.017) (Fig. 4B). None of the other biomarkers were associated with infectious complications.

In Fig. 3B, we report the AUC of the biomarkers associated with infectious complications among COVID-19 patients with abnormal serum CRP values (*n* = 59). Although the AUC among the biomarkers were not statistically different, GDF-15 and suPAR showed higher AUC.

#### Cardiovascular complications

Out of 93 patients, 10 (11%) were complicated with cardiovascular events, including thromboembolism, acute ischemic events, and acute heart failure (Table 1). None of the biomarkers were associated with the cardiovascular complications observed.

## Discussion

In the present study, COVID-19 patients showed an increased inflammatory status, as documented in particular by the high serum levels of GDF-15, PIVKA-II, as well as CRP.

Our cohort was represented by inpatients admitted to hospital because of worsening of the general clinical conditions, but not requiring an intensive care setting. The mean age (67.6 years) was indicative of older adulthood, and no significant difference in gender was present within the cohort studied. In line with the recent literature [20], patients admitted to our division presented with different comorbidities, including arterial hypertension, cardiovascular disease and diabetes, as the most frequent and were treated with ACE inhibitors, angiotensin receptor blockers, statins and anti-diabetic medications, as previously documented in other studies conducted among adults hospitalized for COVID-19 [21]. The median length of stay (22 days) was slightly longer than that reported in other studies, although these data were not obtained in the same setting (non-ICU) and in the same country [22].

By our analyses, we correlated the serum levels of GDF-15, suPAR, PIVKA-II and sdLDL with some clinical characteristics. In particular, we did not find differences in gender, but GDF-15 and suPAR significantly correlated with age, although suPAR did not differ from controls. This may be at least in part determined by an increased inflammatory status frequently observed in older individuals with multiple comorbidities [23, 24]. CRP levels correlated with age in our cohort, and this observation was often reported in previous studies on COVID-19 [25]; also, higher CRP levels were confirmed to be associated with poor outcome in our setting. Importantly, different inflammatory biomarkers were associated with prognosis, not exclusively among COVID-19 patients, but also in other chronic inflammatory diseases, such as cancer, heart failure and other infections [26]. In the ROC curves for poor outcome as well as infectious complications, we did not observe significant differences in the AUC of the biomarkers, likely due to the limited number of the patients considered for this analysis and therefore needing further validation.

Interestingly, GDF-15 levels were associated with the presence of multiple comorbidities, and this was in line with the data from Ramu Adela et al. showing that high GDF-15 plasma levels were associated with multiple chronic conditions, including cardiovascular disease, obesity and chronic kidney disease [27].

Based on the available evidence, hospitalized patients for COVID-19 may worse their clinical conditions especially in terms of respiratory function and cardiovascular compliance and therefore needing more intensive treatments and often developing multiple organs failure with a consequent higher mortality rate [28]. This scenario may develop in a short time frame and physicians should be able to stratify this risk possibly at the beginning of hospitalization and to implement diagnostic and therapeutic strategies to improve clinical response and ultimately the outcomes. To be able to reach this goal, the pathophysiological mechanisms underlying COVID-19 and its more aggressive clinical presentation should be unveiled.

For this reason, considering inflammation as one of the main factors contributing to severe COVID-19, the identification of novel inflammatory biomarkers appears extremely useful and clinically relevant. In this light, among the biomarkers we tested, we found a positive correlation between GDF-15 and length of stay, as well as an association between GDF-15 and worse prognosis, represented by ICU transfer or death.

Our observations are in line with those obtained by others. In fact, Myhre et al. found elevated levels of GDF-15 in most of in-hospital COVID-19 patients, and were associated with higher SARS-CoV2 viremia, hypoxaemia, and worse outcome [12]. In particular, the prognostic role of GDF-15 was more reliable in the early phase of the infection, when compared with other inflammatory biomarkers, including CRP, ferritin, D-dimer, and IL-6 [12]. The role of GDF-15 is still unclear, but GDF-15 seems to be produced, among others, during infections, tissue damage and acts as a metabolic and energy regulator [29].

Robust data indicate that mortality in COVID-19 is frequently determined by superinfections developed during hospital stay, and these observations are common also in non-intensive setting [7, 30]. In particular, the most frequent infections are represented by those of urinary tract [31]. Importantly, superinfections increased the risk of mortality of 50% in COVID-19 patients [30].

In our study, superinfections developed in 40% of the patients and this observation appears different to the data available in some literature where the prevalence is 4–14% based on microbiological tests [32]. However, others indicate the use of antibiotics in COVID-19 in 39% of the patients [33], supporting our results.

Regarding the biomarkers we tested, GDF-15 and suPAR levels were associated with the development of infectious complications, including pulmonary and urinary, as well as sepsis.

As indicated by Lippi et al. who analyzed five studies, GDF-15 concentrations were significantly higher in COVID-19 patients with severe disease [34]. In particular, GDF-15 levels were described to be increased by 78% in patients with severe illness [34]. Interestingly, GDF-15 concentrations remained high throughout ICU stay with a trend towards increase in non-survivors compared with those who survived [35].

Regarding suPAR, non-univocal data are available. In particular, a recent meta-analysis showed that no significant differences for suPAR were found between the severe group compared with mild/moderate group of COVID-19 patients. However, among different biomarkers, patients who died showed higher suPAR concentrations at admission compared to survivors, and frequently developed poor comprehensive clinical prognoses, including infections [36].

In our cohort, PIVKA-II levels correlate with a worse prognosis, but not with superinfections and length of stay. Interestingly, Dofferhoff et al. tested vitamin K status in hospitalized COVID-19 patients, showing that PIVKA-II distribution did not differ among patients with positive or poor outcomes [15]. Moreover, another study found a positive correlation between PIVKA-II (low vitamin K status) and IL-6 levels, but no association was detected with prognosis [37].

In current literature, in the general population, sdLDLs correlate with higher incidence of cardiovascular complications, in particular with venous thromboembolism [38]. The sdLDLs are predictors of cardiovascular residual risk in patients with coronary heart disease on statins treatment, even better than LDL cholesterol [38]. The predictive role of sdLDL and lipoprotein profile in patients with COVID-19 is still controversial [38].

In our cohort, sdLDLs were not associated with poor prognosis, including cardiovascular complications. Since the number of cardiovascular events during hospitalization was small, further clarification on this aspect is needed.

Our study has limitations, including the quite limited number of patients enrolled. Increasing the sample size should be implemented in further studies to confirm our results. PIVKA-II dosage was available in less patients (89% of the sample). We assessed the circulating levels of the biomarkers at the time of admission in the Internal Medicine ward, but their concentrations were not tested at discharge, nor in fully recovered COVID-19 patients nor at the time of negative test for COVID-19, nor when clinical worsening or other complications developed. This information might better clarify the link between the modulation of these biomarkers and prognosis, as well as the effect of the therapies on their concentrations overtime.

In conclusion, our study adds novel information regarding specific biomarkers in COVID-19 in a non-ICU setting. This appears clinically relevant for physicians in order to potentially stratify the risk for a worse prognosis or complications according to their elevated levels, in particular of GDF-15, suPAR and PIVKA-II.

Our data support a crucial role of an enhanced inflammatory status in determining poor outcomes in COVID-19 and additional data should clarify their role as reliable biomarkers to be routinely tested with prognostic value in this setting.

## Data Availability

Not applicable.
